# The Week's Work

**Published:** 1910-12-17

**Authors:** 


### The Week's Work.

### LAY BOARDS AND MEDICAL COMMITTEES.

There is growing up steadily a most regrettable tendency, whose results in the long run can only be disastrous, for lay boards to override the recommendations of their medical committees in reference to new appointments on the staff. We should be the last to assert that such elections should rest solely with the medical committees, and that the lay boards—as representing the subscribers—should be deprived of their powers of Veto, in the cant phrase of the day; but if the power of upsetting the reasoned and reasonable judgments of medical committees is misused, as has so frequently happened lately, a revision of the relations of the recommending and confirming authorities will become inevitable. A year ago comment was offered on an appointment at the Cancer Hospital, when the lay board, for no obvious reason, rejected a perfectly satisfactory suggestion of the medical committee for the filling of a casual vacancy on the junior surgical staff. There was in that case, at least, no suspicion of private influence or personal favouritism about the appointment as made; but there have been at other hospitals in London at least two appointments since then of which this cannot, unfortunately, be said. The latest example is so flagrant that it cannot be passed over in silence.

### A WEST LONDON APPOINTMENT.

At the West London Hospital quite recently a post on the junior staff was advertised in the usual way. The growing reputation of this hospital has been mainly due to the excellence of the medical staff and the way in which the members have developed the post-graduate teaching in connection with the hospital; it is therefore obvious that the staff has a moral right to be consulted about new appointments even greater than in other hospitals. In due course the medical committee, from an excellent entry, selected two of the most promising of the younger generation of consultants, and sent these names to the lay board as the first and second choices for the vacancy. The gentleman whose name stood first of these two would, it is said, have been at once elected had it not been that the Duke of Abercorn interfered with an intimation that another candidate (a fellow-countryman of the Duke) was personally known to him, and proposed that this gentleman be elected. The Duke has always been an active and influential supporter of the hospital, and his *prestige* with the other members of the committee would appear to be such that his wishes were thereupon complied with, to the disgust and amazement of the medical staff. The whole incident is so extraordinary that it would seem incredible were it not that it has more than once been paralleled elsewhere in London. It reflects no credit on the other members of the board that they should allow themselves to be thus led by the nose; and members of the medical committee seem to be contenting themselves with inarticulate and ineffectual grumbling. There is another appointment to be made at the hospital about the time these lines appear; if such backstairs influences are once more allowed to affect the selection, the reputation of the West London will be so seriously prejudiced among the profession that a marked deterioration among the entrants for future appointments may be confidently expected. The continual disregard of excellent candidates recommended by medical committees in favour of those who are personally popular with members of lay boards, or who possess other influence unconnected with their professional achievements, is a development against which it is time to offer a strong protest. It is a matter which King Edward VII. Fund might well take into consideration.

### DUAL HOSPITAL APPOINTMENTS.

We congratulate the Governors of the Leeds General Infirmary on the stand that they have taken with regard to the vexed question of dual appointments. At a special court held on December 8 they unanimously passed the following rule: "That from and after January 1, 1916, members of the full honorary staff shall not hold any other honorary hospital appointments than their appointment at the infirmary. That from and after January 1, 1916, members of the assistant honorary staff shall not hold more than one honorary hospital or dispensary appointment in addition to their appointment at the infirmary, and that such additional appointment must not involve the charge of beds; provided that this rule shall not be held to prevent members of the staff from holding consulting appointments at other hospitals, appointments at the University of Leeds, or appointments to offices in the Territorial Medical Force, or (so long as they are not elected to a different position from that now held by them) from retaining appointments now held by them." This we consider as almost a model regulation. It is not retrospective, it involves no hardship on members of the present staff, who are allowed to retain their outside appointments, and it clearly shows the newcomer that if he accepts an Infirmary post he will be expected to give his whole attention to it. Some such rule is imperatively necessary in other hospital centres. There are many members of hospital staffs in London who hold three or more appointments at so many different hospitals, and, we say it with deliberation, they cannot give equal attention to all their beds: they must neglect one institution, and at any rate they cannot divide their labours so equally that one hospital is not more favoured than another. This is a matter in which the profession as well as hospital workers should be interested. Duplicate appointments beyond a certain limit mean waste of energy, slovenliness, and bad work, which must be prevented at all costs if our institutions wish to retain their prestige.

### HOSPITAL MEDICAL SCHOOLS AND THE KING'S FUND.

Elsewhere in this issue we publish the final reply of the Honorary Secretaries of the King's Fund to the letters emanating from the Metropolitan Radical Federation. The correspondence speaks for itself, and any unprejudiced reader will readily be able to decide which party has the greater claim to sympathy. The Honorary Secretaries' letter, we trust, will close the correspondence, since the constant nagging by outsiders at the work of a fund that tries to do its duty without regard to fads or prejudices is calculated to damage the hospitals by withdrawing public support from it. It is a fair and dignified rejoinder to the allegations made in Mr. Garrity's letter, and its concluding paragraphs are particularly worth noting. We published Mr. Garrity's letter anent the "omission" to print Sir Arthur Bigge's acknowledgment of the Federation's petition without comment, but that does not in the least imply that we shared the Federation's apparent indignation that this acknowledgment was not referred to by the Secretaries of the King's Fund in their replies. The reason given for such "omission" is perfectly sound, and everyone who is aware—as all hospitals are aware—of the practical interest King George takes in the King's Fund must deprecate this barefaced attempt to make it appear that his Majesty is a party to supporting the Federation's attack on the administration of the Fund. In connection with the whole business we may draw the attention of our readers to an able letter from Mr. Sydney Holland which appeared in last week's issue of the *Saturday Review*. Mr. Holland sums up the position of the Federation very clearly and endorses what we have already stated in Week's Work, that the petition of this veritable busy body found inspiration in the anti-vivisectionists' annual attempts to withdraw subscriptions from the hospitals.

### THE REMOVAL OF DANGEROUSLY SICK PATIENTS.

The inquest held last week on an asylum patient at Tooting Bec has drawn public attention to the action of the authorities at the Poplar Workhouse in permitting a patient, who from all accounts was dangerously ill, to be moved a long distance in a brougham. So far as the evidence goes, the workhouse medical authorities seem to have acted in good faith, and to have considered that the man's condition was not so grave as to forbid his removal. On the other hand, the asylum superintendent was of opinion that the long journey in the brougham materially affected the patient for the worse. The pathologist who had been ordered by the Coroner to make an autopsy stated that he found the man had been suffering from heart disease and pulmonary tuberculosis, and added that, in his opinion, death had been accelerated by the trying journey. We need not stop to point out that the pathologist's opinion on the influence of the removal from the workhouse to the asylum is one that, set against the definite statement of the workhouse medical officer, who saw the patient and judged that he was fit to be moved, does not carry much weight. At the same time it is one which undoubtedly impressed the jury. The moral of the whole case is that hospital authorities ought to exercise the greatest caution in sending out of their institutions patients who run the slightest risk of dying outside the hospital. It is this sort of case that comes prominently before the public at Coroners' inquests, and that is at the bottom of every hospital "scandal" of which the lay Press makes so much. Where an ambulance is available, as it appears to have been in this case, it should be requisitioned to take the patient away, in preference to a less comfortable conveyance such as a brougham or cab.

### THE CHRISTMAS APPEAL NUMBER.

The annual Christmas Appeal Number, which we publish this week, is this year of special interest to hospital workers on account of the article dealing with hospital finance. 1910 has been a bad year for the voluntary hospitals, and the elections have not tended to make the public more willing to subscribe to appeals. Every hospital secretary, governor, and friend should make a point of distributing copies of the Appeal Number, marking in blue or red pencil the special paragraphs which deal with the particular institutions in which they are interested. In that way, we venture to think, they may secure new helpers, and at any rate they will be assisting in making known the cause of voluntary charity. The Appeal Number is a practical summary of the needs of hospitals, prefaced by a couple of special articles which deal with the present position. The first of these articles shows how State aid will affect the hospitals—and the rates—and we commend this concise exposition of a difficult subject to everyone who is enamoured of the scheme to municipalise our voluntary charities. The writer shows clearly that State hospitals would mean the levying of an additional rate of over 8s. 6d. for each head of population —man, woman, and child—or, in other words, the increase of local rates from £1 14s. 1d. to over £2 2s. per head per annum of the total population. The Rector of Studland, the Rev. F. Algeo, contributes a well-written and thoughtful article on "Ministering Service," which should be an excellent reminder and stimulus to members of the community at this time of year. Personal service has done much for the hospitals in the past; it will do much for them in the future. May we look to every reader of these pages to help in distributing the Appeal Number, and thereby making known the needs of our charitable institutions?

### THIS WEEK'S DRUG MARKET.

Business in the drug market has been quieter during the week, and very few changes in price have taken place. The value of opium is well maintained, and a small advance in makers' quotations for morphine is not unlikely to take place. Ergot of rye continues to rise in price, and there seems no reason to assume that the highest price has been reached. Menthol is dearer and in fair demand. Castor oil is also still inclined to advance in value. Buchu leaves and jalap are tending cheaper. Gentian is rather dearer.

